# Detection of Artisanal and Small-Scale Gold Mining Activities and Their Transformation Using Earth Observation, Nighttime Light, and Precipitation Data

**DOI:** 10.3390/ijerph182010954

**Published:** 2021-10-18

**Authors:** Satomi Kimijima, Masayuki Sakakibara, Masahiko Nagai

**Affiliations:** 1 Research Institute for Humanity and Nature, Kyoto 603-8047, Japan; sakaki@chikyu.ac.jp; 2 Graduate School of Science & Engineering, Ehime University, Matsuyama 790-8577, Japan; 3 Graduate School of Science and Technology for Innovation, Yamaguchi University, Ube 755-8611, Japan; nagaim@yamaguchi-u.ac.jp

**Keywords:** artisanal and small-scale gold mining, environmental governance, Indonesia, landcover change, mining camp, nighttime light, remote sensing

## Abstract

The rapid growth of artificially constructed mining camps has negatively impacted the camps’ surrounding environment and the informal communities that have developed inside the camps. However, artisanal and small-scale gold mining (ASGM) is generally informal, illegal, and unregulated; thus, transformations of the mining activities and potential social-environmental problems resulting from these changes are not revealed. This study assesses the transformation of mining activities in camp-type ASGM sectors in Gorontalo, Indonesia, during 2014–2020 using remotely sensed data, such as Landsat series, nighttime light, and precipitation data obtained through Google Earth Engine. Results show that the combined growth of the built-up areas increased 4.8-fold, and their annual mean nighttime light increased 3.8-fold during 2014–2019. Furthermore, diverse increases in the sizes of area and nighttime light intensity were identified from the mining camps. Among the studied camps, since 2017, Motomboto camp 3 showed a particularly rapid change in activity regardless of the season of the year. Hence, these approaches are capable of identifying rapid transformations in the mining activities and provide significant insight into the socio-environmental problems originating from the closed and vulnerable camp-based ASGM sector. Our results also contribute to developing rapid and appropriate interventions and strengthening environmental governance.

## 1. Introduction

Rapid growth of artificially constructed mining camps has negatively impacted their surrounding environments and the informal communities that have developed inside them. The communities that have developed in such camps may face severe socio-environmental problems at various levels owing to their informal status. Therefore, detecting such camps and determining their rate of development as well as the transformations of their activities should provide significant insights into and the identification of possible social-environmental problems that originate in these vulnerable mining communities. This may also enable environmental governance to be promoted at various levels.

The artisanal and small-scale gold-mining (ASGM) sector, which is characterized as informal, unregistered, and illegal, is a significant gold-producing sector that uses rudimentary technology at the individual, group, or community levels [1]. In this sector, 70–80% of small-scale miners are informal workers [2]. The United Nations Environment Programme reported that ASGM is the largest employer in gold mining throughout the world, representing approximately 20% (400–600 T/year) of the worldwide gold production and 90% of the global gold-mining workforce, respectively [3]. In the process of gold extraction, mercury is commonly used at the stage of amalgamation, resulting in substantial harmful environmental and health risks owing to mercury pollution [4,5,6], such as mercury emissions into the atmosphere and release into water. Such mercury pollution has mainly been reported in South America, Africa, and Asia [4,7]. Additionally, other health problems—such as silicosis, methyl orthophosphate-oriented poisoning, and various injuries—also occur during the mining process [8]. Despite the high socio-environmental risks, ASGM has been undertaken continuously in more than 80 countries as a tool for poverty alleviation during their socioeconomic development [3,9]. In Indonesia, a continuous growth in ASGM has been observed across the country. Both active and non-active ASGM practices have been located in 93 regencies in 30 out of 34 provinces in Indonesia, estimating more than 1200 hotspots in 2017 [10] with 250,000–300,000 miners [11]. Furthermore, Indonesia’s fastest rise in the number of polluted sites has been reported in the past 20 years at the global level [2]. In Gorontalo province, which shows the fifth highest poverty rate of 25.9% in 2019 [12], many informal mining activities have been widespread even in national park areas, affecting biodiversity and human health [10].

The ASGM sector can be categorized into two types: “travel-type,” in which miners commute daily from their local residences to the mining sites, and “camp-type,” in which miners live and conduct mining activities at the worksites [13]. The camp-type ASGM (hereafter referred to as C-ASGM) sites are artificially constructed, basic settlements—in general, with poor infrastructure—resulting in the formation of an informal society in each camp [13]. The scale and workforce of the ASGM sector has been expanding along with the increase in gold prices since 2000 [14]. A relationship between the increases in ASGM and the high price of gold has been confirmed in the literature [15,16].

Recent research has focused mainly on the environmental and health assessments of mercury pollution originating in the ASGM sector [1,8,17,18,19,20,21,22,23,24]. Several studies have focused on the C-ASGM sites, but they have been limited to point-based, time-cross-sectional analyses [25,26]. Thus, quantitative analyses of the time-series of the transformations of mining activities have not been dealt with in-depth. Furthermore, due to the development of geoinformation technology, several studies have conducted ASGM-related time-series assessments of associated features, such as deforestation, mining-area detection, and geomorphic and hydrological changes [16,22,23,24,25,27], but they have mainly examined travel-type ASGM sites. To investigate the closed C-ASGM sites, [13] recently conducted a quantitative time-series analysis using satellite remote-sensing imagery of the growth of the built-up areas at the C-ASGM sites. However, the authors only captured the growth of the mining camps represented by the built-up areas, and the detailed changes and volumes of mining activities in these camps are not well understood.

However, miners living in the C-ASGM sites may face severe social risks within these communities owing to their informal, illegal, unregulated, and vulnerable natures. Thus, a better understanding of C-ASGM sites is required to reveal hidden, severe social problems. For this reason, [28] investigated the economic outputs of small-scale areas with low economic densities using remote-sensing-based light measurements. Applying such data may provide a key to understanding how remote rural ASGM camps have developed and how their mining activities have been transformed. Thus, tracing the nighttime light (NTL) and weather data associated with the spatial distributions of the built-up areas may provide better indicators of activity transformations in the mining camps located in remote rural areas.

This study primarily assesses the transformation of the ASGM activities during 2014–2020 in Bone Bolango Regency, Gorontalo Province, Indonesia, where active C-ASGM activities have been conducted. Specifically, our objectives were: (1) to assess the built-up areas in the mining camps using the Landsat series and (2) to characterize the mining activities by associating the detected built-up areas with the NTL data obtained using the Visible Infrared Imaging Radiometer Suite (VIIRS) from the National Oceanic and Atmospheric Administration (NOAA) and the Climate Hazards Group InfraRed Precipitation with Station (CHIRPS) data. The results of this study are expected to contribute to identifying potential socio-environmental problems originating in vulnerable mining communities, potentially resulting in the strengthening of environmental governance.

## 2. Materials and Methods

### 2.1. Overall Methodological Workflow

**Figure 1:**
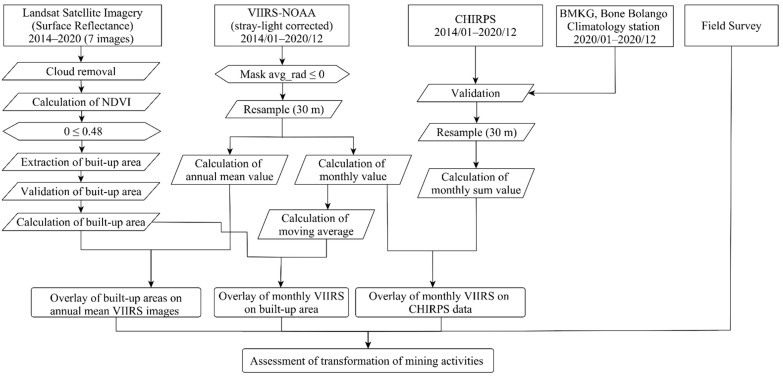
Overall methodology.

Figure 1 shows the methodological workflow used in this study. This workflow employed three main steps to achieve its primary objective of assessing the rapid transformation of ASGM activities. First, the areas built-up in the mining camps during 2014–2020 were identified using Landsat series data. Second, the NTL intensities for those areas were calculated using VIIRS–NOAA data. Third, the amounts of precipitation in those areas were obtained using CHIRPS data. Then, the relationships between the built-up areas was identified, and the volume of NTL and the amount of precipitation were assessed. Together, this evidence enabled us to understand the rapid transformation of the ASGM activities at the mining camps. In this report, we present a discussion based on all the findings described above. The methods utilized in each step are explained in the following sections.

### 2.2. Study Area

North Sulawesi, Indonesia, is a well-mineralized metallogenic region with significant gold mineralization associated with quartz veins in andesite-hosted epithermal settings. The East Suwawa ASGM area is located in Bogani Nani Wartabone National Park, Bone Bolango Regency, approximately 30 km southeast of the city of Gorontalo, Gorontalo Province, Indonesia. This East Suwawa region is categorized as a high-sulfidation epithermal setting containing copper, gold, and silver [29].

The first mining activities in Bone Bolango Regency occurred in the Dutch era (18th century) [30]. Much later, mining activity in the West Motomboto and Tulabolo areas was developed by Tropic Endeavour Indonesia in 1988 [31]. However, these mining sites were closed in 1991 because they overlapped the area being developed into the Bogani Nani Wartabone National Park [31]. The closure of the former mining site triggered the entry of residents to carry out mining activities [31]. In 2013, more than 9000 small-scale miners were reported in the Bogani Nani Wartabone National Park [32].

**Figure 2:**
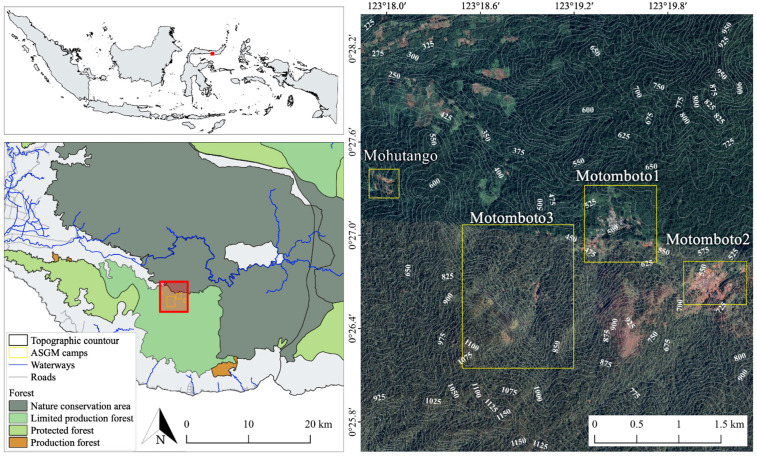
Study area.

In this study, the Mohutango and Motomboto ASGM camps 1, 2, and 3 in East Suwawa in Bone Bolango Regency, Gorontalo Province, Indonesia—each of which utilizes the shaft-based method of mining—are targeted (Figure 2). Those camps are located 4–6 h away from the center of East Suwawa. Access to the camps is very poor; they are only accessible by motorcycle and require the crossing of several rivers in the mountains [13]. The basic settlements in these C-ASGM sites consist of tin roofs covered by tarpaulins, and they are spread across small valleys, forming village-like settlements. All the gold-mining activities are conducted in these simple camps using 24-h shift operations [13].

### 2.3. Satellite Imagery

Atmospherically corrected, cloud-free Landsat data from the Enhanced Thematic Mapper Plus (ETM+) and Operational Land Imager (OLI)’s surface-reflectance products, available from the United States Geological Survey, together with the VIIRS–NOAA and CHIRPS data products, were used. Those datasets are available in Google Earth Engine, and they can be used to extract and calculate time-series of the built-up areas, the NTL intensities, and the amounts of precipitation. Therefore, the Landsat series from 2014 to 2020, which has a ground resolution of 30 m in the World Geodetic System 84 (WGS84) geographic-coordinate reference system and applies the cloud-removal function, was utilized to extract the built-up areas. Furthermore, VIIRS (stray-light-corrected)–NOAA datasets acquired during 2014–2020 were masked, with the NTL values masked to be greater than or equal to 0. Then, annual mean and monthly values were calculated by applying resampling to a spatial resolution of 30 m. The CHIRPS datasets acquired for 2014–2020 were used to calculate monthly-sum values by resampling them to the same scale. Finally, the resulting data for the built-up areas, annual mean NTL, monthly NTL, and monthly precipitation were overlaid.

In previous studies, the mining areas in Bone Bolango Regency were estimated to cover a total of 0.62 km^2^ in 2012 [26]. However, a continuous expansion of the built-up areas in the camps that employ 24-h shift operations has since been reported [13]. Hence, the long-term trends in ASGM camps could be observed from satellite imagery even with a 30-m ground resolution. The main specifications of the databases used in this study are summarized in Table 1.

### 2.4. Extraction of Built-Up Areas, NTL, and Precipitation Data

Satellite-based observational data—such as Landsat, VIIRS, and CHIRPS—acquired in 2014–2020 were used. Because the transformation of ASGM activities in remote rural areas is associated with the miners living at the worksites, a combination of the growth of the built-up areas and associated changes in the NTL can provide significant indicators for assessing the detailed activity in the camps. Furthermore, as described in Section 2.2, the basic settlements in the studied C-ASGM sites are made of poor materials; thus, their mining activities have higher sensitivity to weather conditions. To clarify their changes, we further assessed them along with the changes in precipitation amount.

In this study, the built-up areas were defined based on their physical aspects, such as a built-up environment consisting mostly of human-constructed elements [33]. A number of spectral indices, together with human visual interpretation [33,34,35,36,37,38,39,40,41], were employed to detect built-up areas using remote-sensing technology. Previous studies found that the Normalized Difference Built-up Index (NDBI) [42,43] and the Urban Index (UI) [44,45] had high sensitivities for retrieving built-up areas; however, these have been employed mainly in urban studies. As the NDBI and UI are incapable of separating built-up areas from bare land effectively [35], separating the two in rural areas can be expected to be more complicated. Therefore, in this study, the Normalized Difference Vegetation Index (NDVI) was applied, as employed by [13,46], to detect remote rural mining areas over long timescales. The value of NDVI in the built-up areas was calculated using Equation (1):

*NDVI* = (*NIR* − *Red*)/(*NIR* + *Red*) (1)

NDVI, which ranges from −1 to +1, has a high value for denser vegetation, while it is lower for desert or non-vegetation areas [47]. In this study, the NDVI was further restricted to the range 0 ≤ NDVI ≤ 0.48 in order to exclude vegetated areas on the land surface from the built-up areas. This threshold value was determined based on comparisons to the accuracy levels for high-resolution satellite data. In this way, the results were visualized in time-series. A hundred points were randomly selected within the study area, and the accuracy of the results was assessed using a high-resolution image obtained on 8 February 2017 from Google Earth Pro. Because images were not available on the same date for which the Landsat imagery had been acquired, images acquired on the closest date, 24 April 2017, were used. In this study, the validated accuracy was applied to all classification results owing to the unavailability of reference data.

NTL data were acquired from the VIIRS, representing radiance values from −1.5 to 193,564.92, for the period 2014–2020. The negative radiance is generated by the airglow effect in unpopulated regions, where the probability of illumination is zero or very low [48]. For this study, values less than 0 were excluded from the whole dataset. After summarizing the monthly radiance values, a 12-month moving average was calculated for 2014–2020. Meanwhile, an annual mean radiance value was calculated per year to generate a time-series of annual maps, which were overlaid against the detected built-up areas.

Precipitation data was acquired from CHIRPS for the period 2014–2020. Before the CHIRPS data were analyzed, data consistency was evaluated using reference data observed at Bone Bolango climatology station provided by the Indonesian Agency for Meteorology, Climatology and Geophysics (BMKG) [49]. Precipitation data provide by CHIRPS were accumulated in the form of monthly data to match the monthly data provided by BMKG. After the data validation, the sums of the amounts of precipitation by month were calculated for each camp. These results were graphed together with the detected monthly NTL.

### 2.5. Investigation of ASGM Camps

Field observation was conducted on 6 February 2020 to investigate the ASGM camps. Additionally, interviews were conducted with key informant miners on the worksites.

## 3. Results

### 3.1. Growth of Built-Up Areas in the Mining Camps

**Figure 3:**
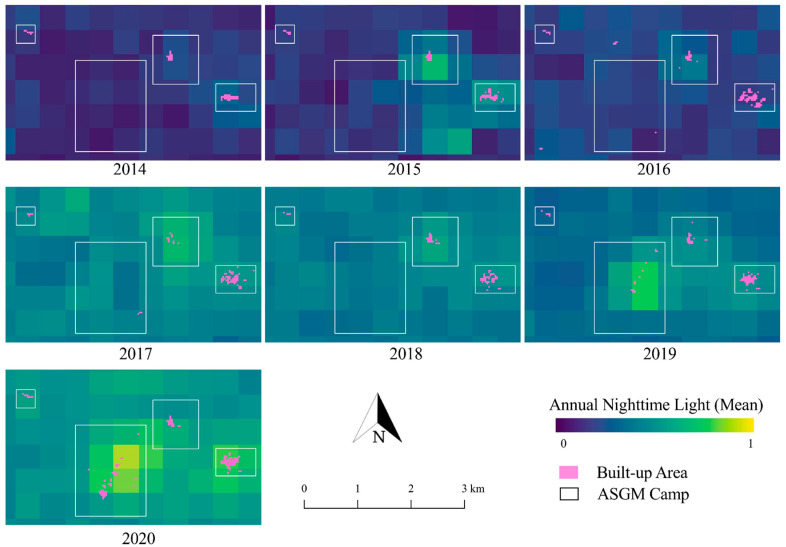
Overlay of built-up areas on annual mean NTL images in time-series.

To detect the land-cover changes surrounding the ASGM camps during the period of 2014–2020, the calculated NDVI was primarily used. Using these NDVI results, the built-up areas in the ASGM camps were calculated with an accuracy of 99%, as described in Section 2.4. Figure 3 shows how the built-up areas have developed over time in the camps. The built-up areas in the Mohutango and Motomboto ASGM camps 1 and 2 were identified beginning early 2014, and camp 3 was identified early in 2016, and the camps showed various types of growth. The growth of all the built-up areas combined exhibited a 4.8-fold increase during 2014–2020. While the Mohutango and Motomboto ASGM camp 1 remained similar in extent from 2014 to 2020, the Motomboto camps 2 and 3 developed substantially in extent in 2015 and 2019, respectively. Among these mining camps, the growth of camp 3 is clearly distinguishable from the cases mentioned above, showing continuous and rapid annual growth of the built-up areas to the southern part. While the growth of the Mohutango camp and Motomboto camp 1 showed only 1.1- and 1.4-fold increases, respectively, during 2014–2020, and camp 2 showed a 1.2-fold increase during 2015–2020, camp 3 showed a remarkable 23.1-fold increase during 2016–2020. Through the field observations, we confirmed that the identified built-up areas were either residences of miners or settlements for mining activities where trommel machines and pools for immersing the materials were placed. Furthermore, the ASGM activity in this area has rapidly increased since 2017 after the gold price increased (according to interview with a local miner).

### 3.2. Relationship between Built-Up Areas and NTL Intensity in the Mining Camps

The built-up areas were further overlaid against the corresponding annual mean NTL images as an indicator of the volume of mining activity in time-series (Figure 3). The total annual mean NTL showed a 3.8-fold increase during 2014–2020, while the individual Mohutango and Motomboto camps 1, 2, and 3 showed 3.0-, 2.8-, 2.6-, and 5.4-fold increases, respectively. The highest NTL increase occurred in mining camp 3, where the rapid growth of built-up areas was observed (Section 3.1). On the contrary, the Mohutango and Motomboto camps 1 and 2, which were already identified in 2014, showed lower growths of NTL intensity in comparison to Motomboto camp 3. Based on our field observations, electricity was generated using diesel generators and distributed across the camp (as reported by a local miner).

**Figure 4:**
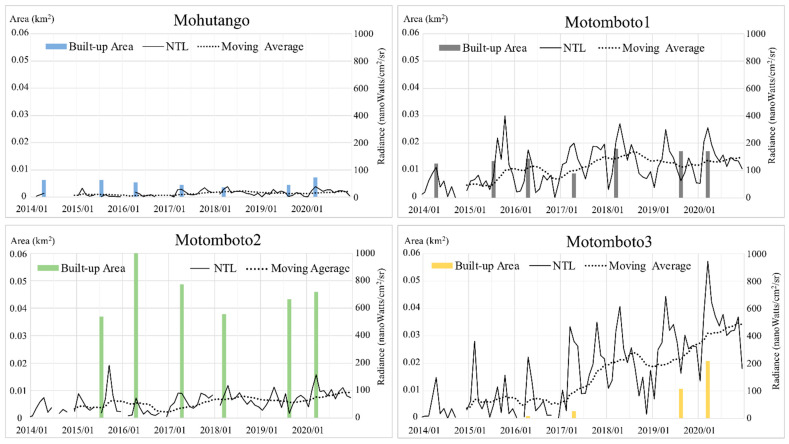
Built-up areas and monthly NTL intensities for the ASGM camps.

To deepen our understanding of the mining activities in the camps represented by the NTL intensity, the monthly NTL intensity and the 12-month moving average of NTL were graphed against the built-up areas (Figure 4). Even though the NTL intensities vary by camps, the study found a similar trend of the NTL intensity by season. For example, lower NTL intensities were found in the rainy seasons—i.e., during April–June and November–December—while higher intensities were found in the other months, during the dry seasons. There was no significant change in the maximum values in the Mohutango and Motomboto camps 1 and 2; however, the NTL from Motomboto camp 3 showed a 2.0-fold increase for 2014–2020. Furthermore, notable increases in the NTL were found in Motomboto camp 3 even in the rainy season, along with the growth of built-up areas since 2017. The 12-month moving average for Motomboto camp 3 also showed a continuous increase.

### 3.3. Relationship between the NLT Intensity and Precipitation by Month

**Figure 5:**
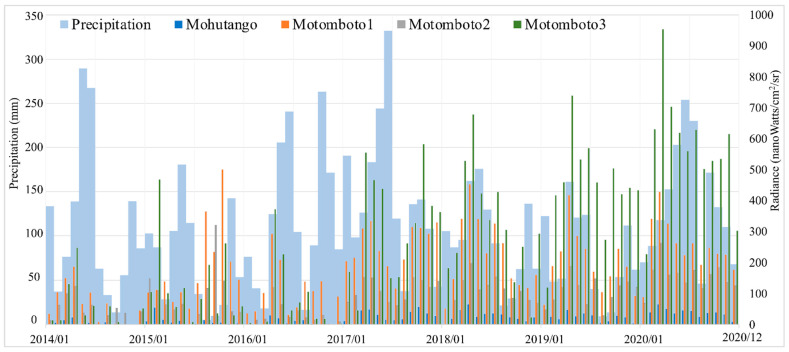
Monthly precipitation (sum) and NTL intensity by mining camps.

As described in Section 2.2, the basic settlements in the studied C-ASGM sites are made of tin roofs covered by tarpaulins; consequently, their mining activities may have higher sensitivity to weather conditions. We therefore expanded our analysis of the NTL intensity described in Section 3.1 by associating it with the amount of precipitation, with a correlation of 89%. As described above, higher NTL intensities have been observed since 2017, even in the rainy season, especially in Motomboto camp 3; thus, we summarized the monthly precipitation and NTL intensities by camp in a graph (Figure 5). The annual precipitation in 2014–2020 was 1333, 887, 1437, 1760, 1170, 918, and 1643 mm, respectively. Even though the annual amount of precipitation varied, the NTL volumes in 2020 were the highest during the entire study period. Despite the increase in the volume of precipitation, the NTL intensity increased during the rainy season even in the simple settlements, especially after 2017.

## 4. Discussion and Limitations

### 4.1. Discussion

We studied the rapid transformation of activities in the C-ASGM sector from 2014–2020 using time-series associated with the built-up areas, the NTL, and precipitation data. A quantitative time-series analysis of the artificially constructed C-ASGM sectors can help to achieve better understanding of the rate of development of such mining activities and their transformations across time. Detecting such rapid transformations can provide significant insights into hidden, severe social problems inside these vulnerable mining communities, potentially resulting in the strengthening of environmental governance at various levels.

By combining the extraction of indicators of the growth of the built-up areas of C-ASGMs, the NTL intensity as an indicator of the mining activities, and the precipitation data, this study demonstrated the transformations of the mining activities undertaken in the C-ASGM sectors over a significant fraction of a decade (Figure 3, Figure 4 and Figure 5). Using a quantitative analysis over the time, this study detected the various forms of built-up areas and NTL intensities in the mining camps. For example, Motomboto camp 3 was identified in 2016, and it showed a more rapid and extensive growth of the built-up areas and the NTL intensity than the other camps during 2016–2020, with 23.1- and 2.0-fold increases, respectively, as described in Section 3.1. We found notable increases in Motomboto camp 3 since 2017, which is in accordance with a previous study [13]. As the study sites are remote, rural C-ASGMs, a significant source of the growth identified in this area may be due to large influxes of miners from neighboring regions, such as Bolaang Mongondow and Minahasa in North Sulawesi [32]. This huge entry may have been encouraged by weak regulations resulting from the informal, illegal, and closed nature of this sector [9]; limited government resources and administrative capacity to provide adequate technical assistance or enforce compliance [50]; and the remote locations of the mining sites [27], which could further result in large socioeconomic problems.

The transformation of the working pattern was also identified since 2017 regardless of the season. This may imply that local villagers near the mining sites previously engaged in the mining activity as an additional income-generating activity during the agricultural off-season. However, the increased influx of miners from neighboring regions that has occurred since 2017 appears to have resulted in workers staying at the mining camps continuously, becoming occupational miners throughout the year. As discussed above, [13] previously revealed the expansion of the C-ASGM sector; however, the increase of NTL, in association with the precipitation data, enables the further identification of the detailed volumes of and changes in the hidden mining activities.

The C-ASGM sector can operate successfully owing to its high productivity of gold. Despite its status as an informal sector, large influxes of miners have entered the camps continuously, resulting in their rapid growth. These influxes of large populations into artificially constructed spaces, which lack fundamental infrastructures and systems, definitely cause and accelerate socioeconomic and environmental problems relating to children, family, education, health, sexual health, sanitation, garbage, and water usage, as reported elsewhere as well [51,52,53,54]. However, their informal and illegal status limits the power of law over and control of the camps, resulting in severe situations at some camps. In particular, this may be the case for the rapidly growing C-ASGM sector, as has been observed at Motomboto camp 3. Thus, detection of such rapidly developing, hidden C-ASGM sectors can definitely contribute to strengthening environmental governance by attracting and involving various stakeholders at various levels.

Many of ASGM-related assessments are limited to a particular discipline; thus, interdisciplinary researches involving health and environmental impact assessments will be carried out in different regions to prove the effectiveness of remote sensing analysis in the future.

### 4.2. Limitations

The results of this study have some limitations associated with the quality of the input data. First, the presence of small negative-radiance values caused by the airglow effect in uninhabited regions [48] can lead to underestimates of the NTL intensity. Second, differences in the spatial resolution of the utilized datasets results in mixed pixels, which can cause the overestimation or miscalculation of factors such as built-up areas and NTL intensities. Third, the methodology used in this study is applicable only to similar mining sectors that employ 24-h operations.

## 5. Conclusions

The rapid growth of artificially constructed mining camps has negatively impacted the environment surrounding them and the informal societies that have formed inside them. In this study, the transformations of the ASGM activities in Bone Bolango Regency, Gorontalo Province, Indonesia, were assessed using remote-sensing data. The results presented herein show that the growth of the built-up areas and annual mean NTL experienced 4.8- and 3.8-fold increases, respectively, during 2014–2020. In particular, rapid changes in the working patterns were found in Motomboto camp 3 regardless of the season. Therefore, it can be concluded that transformations of the activities undertaken in the closed C-ASGM sites can be determined by combining observations of the built-up areas and NTL in the mining camps with the precipitation volumes. These results extend our understanding of the transformations of mining activities in the hidden C-ASGM sectors and provide significant insight into the potential for social problems that can occur in vulnerable informal mining communities. These findings are expected to assist in developing rapid and appropriate interventions for strengthening environmental governance by involving various stakeholders.

## Figures and Tables

**Table 1:** Main specification of satellite imagery in the study.

Satellite	Acquisition Date	Temporal Resolution	Spatial Resolution
NOAA VIIRS	2014.01.01–2020.12.31	Monthly	15 arc seconds
CHIRPS	2014.01.01–2020.12.31	Daily	0.05 degrees
Landsat 7 ETM+	2014.04.24	16 days	30 m
Landsat 8 OLI	2015.07.08	16 days	30 m
	2016.04.05		
	2017.04.24		
	2018.03.10		
	2019.08.04		
	2020.03.15		

## Data Availability

Not applicable.
